# Hemispheric Asymmetries in Repetition Enhancement and Suppression Effects in the Newborn Brain

**DOI:** 10.1371/journal.pone.0140160

**Published:** 2015-10-20

**Authors:** Camillia Bouchon, Thierry Nazzi, Judit Gervain

**Affiliations:** 1 Université Paris Descartes, Sorbonne Paris Cité, Paris, France; 2 CNRS–Laboratoire de Psychologie de la Perception (UMR 8242), Paris, France; 3 Center for Brain and Cognition, Universitat Pompeu Fabra, Barcelona, Spain; Rutgers University, UNITED STATES

## Abstract

**Background:**

The repeated presentation of stimuli typically attenuates neural responses (repetition suppression) or, less commonly, increases them (repetition enhancement) when stimuli are highly complex, degraded or presented under noisy conditions. In adult functional neuroimaging research, these repetition effects are considered as neural correlates of habituation. The development and respective functional significance of these effects in infancy remain largely unknown.

**Objective:**

This study investigates repetition effects in newborns using functional near-infrared spectroscopy, and specifically the role of stimulus complexity in evoking a repetition enhancement vs. a repetition suppression response, following up on Gervain et al. (2008). In that study, abstract rule-learning was found at birth in cortical areas specific to speech processing, as evidenced by a left-lateralized repetition enhancement of the hemodynamic response to highly variable speech sequences conforming to a repetition-based ABB artificial grammar, but not to a random ABC grammar.

**Methods:**

Here, the same paradigm was used to investigate how simpler stimuli (12 different sequences per condition as opposed to 140), and simpler presentation conditions (blocked rather than interleaved) would influence repetition effects at birth.

**Results:**

Results revealed that the two grammars elicited different dynamics in the two hemispheres. In left fronto-temporal areas, we reproduce the early perceptual discrimination of the two grammars, with ABB giving rise to a greater response at the beginning of the experiment than ABC. In addition, the ABC grammar evoked a repetition enhancement effect over time, whereas a stable response was found for the ABB grammar. Right fronto-temporal areas showed neither initial discrimination, nor change over time to either pattern.

**Conclusion:**

Taken together with Gervain et al. (2008), this is the first evidence that manipulating methodological factors influences the presence or absence of neural repetition enhancement effects in newborns and stimulus variability appears a particularly important factor. Further, this temporal modulation is restricted to the left hemisphere, confirming its specialization for learning linguistic regularities from birth.

## Introduction

Neural repetition effects are increases (repetition enhancement) or decreases (repetition suppression) of the neural activity observed over time, in response to repeated presentations of similar (or identical) stimuli. They were found at different levels of brain organization, using different measurement techniques and constitute the neural correlates of various learning mechanisms. Single-neuron recordings [1] directly show the reduced firing rate of neurons in response to stimulus repetition [2]. For large populations of neurons, such effects have been observed in electrophysiological measures using EEG over the scalp [3,4, 5], as well as in hemodynamic correlates of brain activity, such as blood oxygen level-dependent (BOLD) response measured in functional magnetic resonance imaging (fMRI) [6,7 for recent reviews] and near-infrared spectroscopy (NIRS; [8]).

The exact relationship between neural repetition effects observed at the hemodynamic level and the cognitive interpretations that can be drawn are complex and still debated. In adult fMRI studies, such repetition effects are sufficiently documented to be used as signatures of different cognitive processes including discrimination or learning, independently of the direction (enhancement or suppression) of change. By contrast, neural repetition effects are much less understood in infancy.

Specifically, recent studies using near-infrared spectroscopy with neonates and young infants have found seemingly contradictory effects in response to repeated stimuli. Some studies [8,9] have used paradigms in which identical stimuli, linguistic and visual respectively, were repeatedly presented to young infants. They obtained a classical repetition suppression effect, whereby the hemodynamic response decreased gradually over repeated stimulus presentation. Moreover, after several repeated presentations of the same stimulus, a novel stimulus was presented, and discrimination between the initial and the novel stimulus was inferred from an increase in the hemodynamic response after the switch in stimuli. By contrast, Gervain et al. (2008) (later on referred to as [10]) found greater activity in response to an abstract structural regularity (ABB: “mubaba”, “penana” etc.) as compared to otherwise similar random sequences (ABC: “mubage”, “penaku” etc.), and that this differential hemodynamic response to ABB further increased over the course of the study, i.e. to repeated presentations of the ABB structure. Can these seemingly contradictory repetition effects be explained and reconciled? More specifically, can a repetition enhancement effect, as observed in [10] in newborns using NIRS, be interpreted as a neural marker of habituation or learning in the same way as the more commonly observed repetition suppression effect? The current study aims to address these questions, thus contributing to a better understanding of the properties of the hemodynamic response in young infants.

In adults, fMRI experiments have widely used adaptation paradigms to probe brain regions specifically primed by repetitions of stimuli, such as visual objects and their geometric properties [11,12], numbers [13], as well as auditory and speech stimuli [14,15,16,17] and observed neural repetition suppression and enhancement effects (respectively decreases and increases to repetitions of identical stimuli as compared to varying stimuli). Repetition suppression in adult fMRI studies is typically considered to be the hemodynamic signature of neuronal adaptation to fully processed stimuli [7–18], while repetition enhancement typically occurs when the repeated stimulus is initially unfamiliar [19] unattended [20] or shown under conditions affecting stimulus quality: short exposure time [21], low visual stimulus quality or visibility [22,23], low discriminability [24], or degraded stimulus [23–25,26]. These neural repetition effects are thus often used as neural correlates of cognitive functions in various modalities, including language processing [27]. Interestingly, an fMRI study with 3-month-old infants observed an increase of activity, i.e. repetition *enhancement*, in the left inferior frontal region (especially Broca’s area) in response to sentences repeated every 14 seconds [28], whereas adult data by the same authors had shown a repetition *suppression* effect in broad temporal and inferior frontal regions [17] under the same conditions. Independently of the direction of the repetition effect, the authors interpreted both as evidence of memory function in the two populations.

Recently, it has been suggested that repetition effects found in the developing brain should also be considered as indicators of learning and may be interpreted as neural habituation [29]. According to this hypothesis, neural repetition effects could be signatures of habituation to repetitive stimuli and therefore evidence for learning in the infant brain, similarly to habituation effects observed at the behavioral level [30]. In this perspective, we hypothesize that an important factor that might have contributed to the differing directions of the repetition effects observed in the aforementioned NIRS studies (enhancement in [10] vs. suppression in [8]-[9]) is stimulus variability/complexity. Indeed, stimulus complexity is known to impact infants’ habituation curves for behavioral measures (e.g. sucking rate, looking time etc., [30]), with more complex, less predictable stimuli requiring more exposure to achieve habituation. Furthermore, in speech perception and language acquisition, introducing variation in the input was found to help rule extraction [31], while increasing redundancy (i.e. the number of repetitions of the same item in the input) led to rote memorization [32]. Therefore, as different levels of variability in the stimuli yield different learning processes, they should also influence the direction of the repetition effect evoked, i.e. repetition suppression vs. enhancement. The current study seeks to address this hypothesis, and thus to contribute to our understanding of the link between repetition effects and their cognitive/functional interpretations in the infant hemodynamic response at birth.

Concretely, we suggest that it is the high variability of the stimuli used in [10] as compared to [8] and [9] that might underlie the opposite repetition effects found in these studies. Indeed, while the latter two studies contained several subsequent repetitions of the same stimuli, in [10], replicated subsequently with different NIRS systems [33], in different infant populations (in 7- and 9-month-old infants: [34]) and even behaviorally [35], stimulus variability was much greater. Both the ABB and the ABC condition involved 140 different syllable sequences. In fact, the same syllable sequence was never repeated in the experiment, and only the underlying ABB or ABC structure was repeated. Following the analogy between repetition effects and habituation proposed in [29], the left lateralized repetition enhancement effect in the NIRS signal observed in [10] might thus indicate a novelty effect, i.e. not fully completed learning, for the ABB grammar. According to this interpretation, the high variability of the linguistic material used might explain why an *enhanced*, and not a suppressed neural response, was observed. This repetition enhancement effect suggested that full habituation to the ABB structure had not been reached during the time course of the study. Consequently, decreasing the variability in the stimulus material should favor complete learning of the ABB rule that would be indicated by a cortical repetition suppression effect over time. This hypothesis is also supported by the facilitation model proposed by Grill-Spector et al. (2006) which suggested that the greater the number of repetitions in the input (the lesser variability in the material), the faster and the earlier the memory trace formation is detected, diminishing the hemodynamic response until no activation is found.

Therefore the current study tested newborns’ detection and learning of the ABB vs. ABC patterns in trisyllabic sequences, as in [10], but with two substantial differences in the construction of the stimulus material, which might favor more complete learning. First, in order to increase levels of cortical activation and thus improve statistical power in the time course analysis, we did not interleave blocks of ABB and ABC sequences as in [10], but rather presented each condition separately [36,37,38,39], and we reduced the total duration of exposure from 14 blocks of 10 sequences in [10], to 12 blocks of 6 sequences, per condition. Second, and more importantly, the current material contained only a limited number of different sequences to increase the probability of habituation to the material (12 different sequences per condition, as opposed to 140 in [10]; see details below).

Note that in the current experiment, as in [10] and in other studies investigating rule extraction from speech following Marcus et al.’s (1999) seminal study, ABB structures, having identical 2^nd^ and 3^rd^ syllables, are compared to ABC structures. Differential responses between the two structures have been found immediately at the onset of presentation, as in [10]. This differential response has been interpreted as a low-level, automatic mechanism detecting identity or sameness. Furthermore, in [10], the difference between the responses to the two structures increased over the course of the experiment, giving rise to a repetition enhancement effect. This was induced by the repeated presentation of the sequences unfolding in the time, over the course of the experiment. The neural responses observed for this repeated presentation, as defined by our paradigm, allows us to tap onto different cognitive mechanisms involved in learning (e.g., rule extraction or item-based memorization).

NIRS is an ideal tool to address the issue of neural habituation, as it offers the possibility to measure the metabolic correlates of neural activity in a completely non-invasive fashion, well suited for newborns and young infants [34–40,41,42]. Specifically, it measures the cerebral blood oxygenation changes associated to neural activity [43]. Here we use the most common multiple-channels NIRS system is the optical topography (OT) system. Pairing sources and detectors (optodes) to form measurement channels at the surface of the scalp, it provides a two-dimensional sampling of the cortical surface. The source–detector distance (S-D distance) determines the depth of penetration, and the spatial resolution. Critically in newborns and young infants, the optimal S-D distance providing a satisfactory trade-off between depth of penetration and spatial resolution, which yielded replicable results, is between 2.5 and 5 cm [34]. As a result, NIRS only probes the surface of the cortex, with a penetration depth of approximately 10–15mm into the neonate brain [34–44]. It is thus inappropriate for testing structures lying deep in the brain, e.g. those involved in memory and emotions, but it has proven very successful in investigating language areas in the cortex, in particular in infants [42–45,46,47].

## Materials and Methods

NIRS is a neuroimaging technique that measures the hemodynamic correlates of neural activity, using near infra-red light shed at the scalp. Changes in oxy- and deoxy-hemoglobin concentration are calculated from the differential photon absorption properties of oxygenated and deoxygenated hemoglobin [48–49]. fNIRS has been widely used in developmental cognitive neuroscience over the last two decades due to its advantages over fMRI (non-invasiveness, relative tolerance to head movements, absence of acoustic noise, relative affordability etc.), as well as precise spatial localization than EEG [50].

### Participants

Twenty-four full-term, healthy neonates (ranging in age from 1 to 3 days, mean age: 1.8 days; Apgar score 10 minutes after birth: 10; 13 girls; mean head circumference = 34.5 cm) born to French-speaking mothers were included in the analyses. An additional 23 infants were tested, but their data were excluded due to the infant becoming awake or fussy and failing to complete the procedure (11) including 7 non-starter babies who cried when the cap was placed on their heads, equipment failure (2), or insufficient analyzable data (10) due to 6 babies with unusually large amounts of dark hair and one with smaller head size. Data were considered insufficient when more than 50% of the channels had less than 50% analyzable blocks. All infants were tested while asleep. All parents of infants gave informed written consent prior to beginning the experiment. The CERES (Comité d’Evaluation des Projets de Recherche en Santé) has approved this study under the approval nr. 2011-13-2.

### Stimulus material

As in Experiment 1 of [10], newborns were exposed to a repetition-based ABB artificial grammar (e.g. “mulele”, “junana”) and random ABC control grammar (e.g. “mulevi”, “junary”). Both grammars generated trisyllabic sequences and the two grammars were matched on all nonstructural properties: syllabic repertoire (Table 1); frequency of the A, B, and C syllables; phonological characteristics; flat prosody; and transitional probabilities between syllables. Sequences were synthetized using the fr4 female voice of the MBROLA diphone database [51], in a monotonous pitch of 200 Hz, with the same length for all phonemes (150ms).

**Table 1:** Details on the stimuli used in Gervain et al. (2008) experiment 1 and in the current experiment.

	CV inventory	Number of different sequences generated by CV combinations	Within–word TPs between syllables Mean (SD)	Total number of sequences presented in experiment	Sequence frequency in experiment
**Experiment 1 (Gervain et al., 2008)**	20 CV syllables:12 Cs, 5 Vs	280 CVCVCVs: 140 ABB and 140 ABC	0.10 (0.07)	280 (14 blocks of 10 sequences per condition)	Exactly 1 time
**Current experiment**	12 CV syllables (*li le ry ra mu mo ny na vi ʒu ʒo ve*), 6 Cs and 6 Vs	24 CVCVCVs: 12 ABB and 12 ABC	0.33 (0.12)	144 (12 blocks of 6 sequences per condition)	Exactly 6 times, in 6 different blocks

However, unlike in [10], the current experiment used simpler material. Specifically, we used 12, rather than 20 different syllables, made up of an equal number of consonants and vowels: 6 different consonants (Cs) and 6 different vowels (Vs), rather than of 12 Cs and 5Vs. Furthermore, these syllables were combined to yield 12, rather than 140 different trisyllabic sequences per grammar. As a result, in the current experiment the same sequence was presented multiple times during the session (6 times exactly), introducing redundancy in the sequences presented, which was completely absent in [10]. The mean transitional probability (TP) between adjacent syllable pairs was 0.33 for a total number of 48 combinations here, rather than a mean TP of 0.10 for 254 different combinations in [10]. Thus the current material’s increased predictability should favor its learnability. Details are summarized in Table 1.

### Procedure

**Fig 1:**
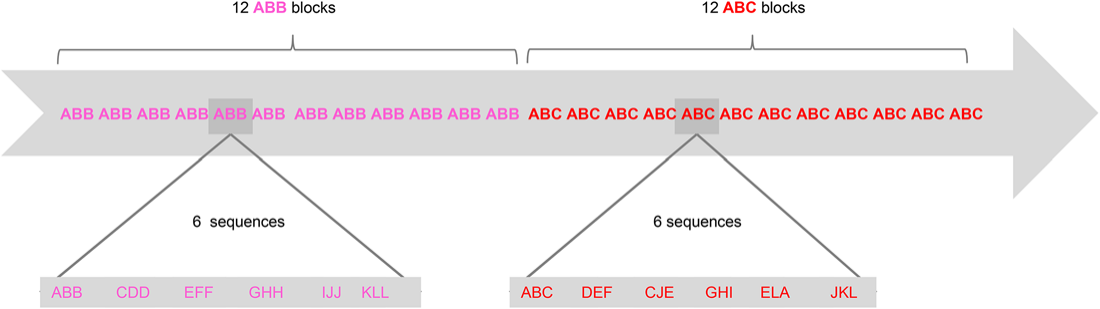
The block design used in the current study. Condition order was randomized and counter-balanced across infants. Letters from A to L represent the 12 syllables extracted from the CV inventory.

Neonates were tested in a local maternity hospital, in a silent experimental room, while asleep or at rest in a bassinet. Each infant was presented with 24 blocks of stimuli. Within a block, the six sequences, which lasted 0.9s each, were separated by brief inter-stimulus pauses of variable length (0.5 or 1.5 s), yielding a total block duration of 9.9 s or 10.9 s. Blocks were also separated by inter-block pauses of 20 or 25 s to avoid inducing phase-locked brain responses. The total testing time for each infant was 14.6 min. The block design used is presented in Fig 1. All participants were presented with both the ABB and ABC conditions. Condition order was randomized and counter-balanced across participants, such that half of the participants were presented with the ABB condition first, the other half with the ABC condition first.

**Fig 2:**
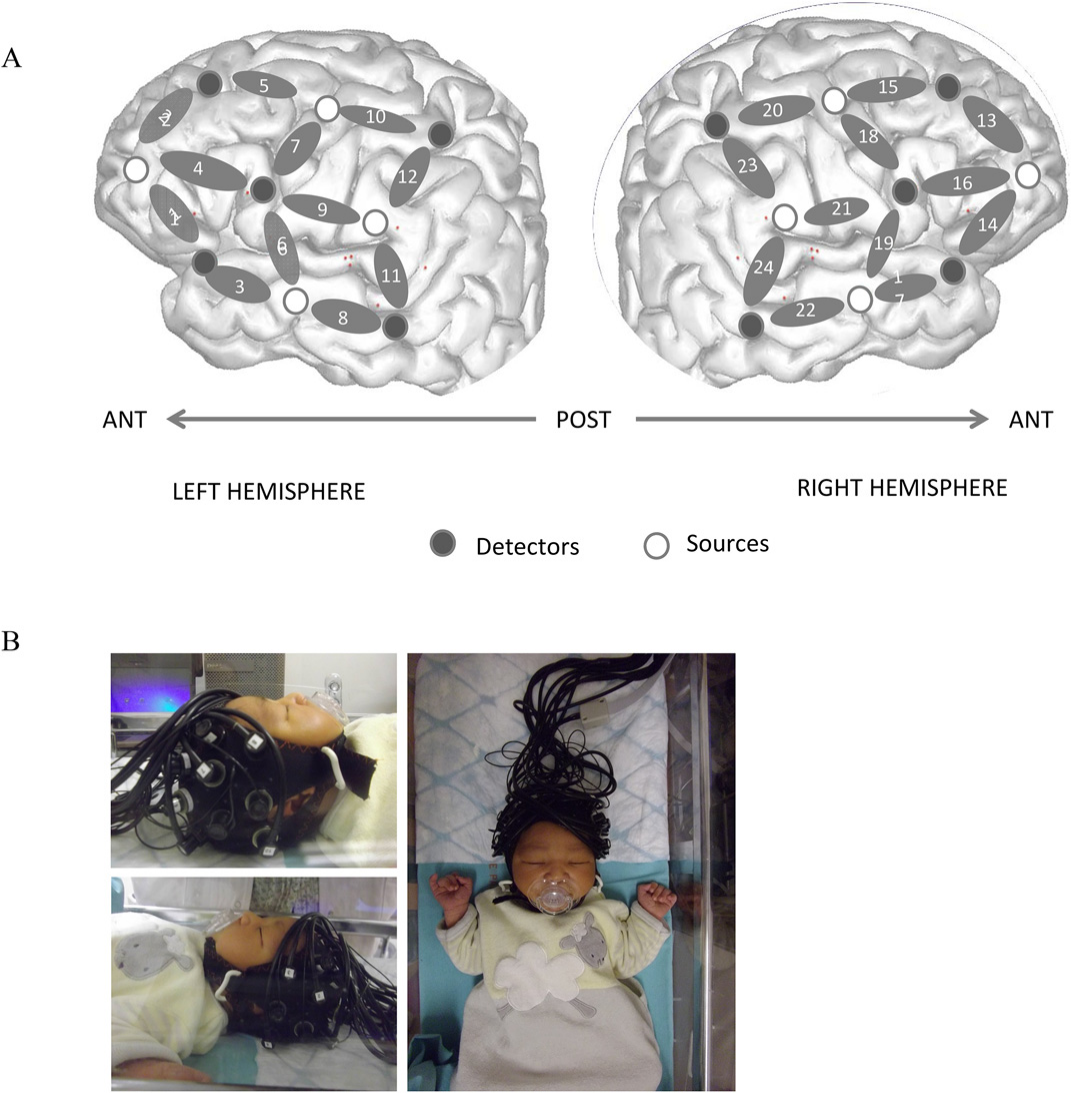
(A) Configuration of probe sets overlaid on an infant skull, superimposed on a schematic infant brain for illustrative purposes and should not be interpreted as a precise mapping of probe channels to ROIs. (B) Picture of a newborn participant with optodes placed upon the head.

Optical imaging was performed with an optical topography imager (*NIRScout* 816, *NIRx Medizintechnik GmbH*, Berlin, Germany) using pulsated LED sequential illumination (5 mW) with two wavelengths of 760 nm and 850 nm to record the NIRS signal at a 10.417 Hz sampling rate. Four LED sources were placed on each side of the head in analogous positions, and were illuminated sequentially. They were coupled with 5 detectors on each side of the head. The configuration of the 24 channels (12 per hemisphere) created with the 4 sources and 5 detectors per hemisphere is shown in Fig 2A. To keep the optodes in place with a standard 3 cm separation, they were embedded in a cotton cap (*Easycap*) of 36 cm diameter (Fig 2B). A MacBook laptop running PsyScope controlled the experiment, played the language stimuli and sent markers to the NIRS machine. The stimuli were played through two speakers approximately 1 m from the infants’ head.

### Data analysis

Analyses were conducted on oxyHb and deoxyHb in a time window between 0 and approximately 30 s after stimulus onset to capture the full time course of the hemodynamic response in each block [10].

Data were band-pass filtered between 0.01 and 0.7 Hz to remove low-frequency noise (i.e., slow drifts in Hb concentrations) as well as high frequency noise (i.e., heartbeat). Movement artifacts were removed by identifying block-channel pairs in which a change in concentration greater than 0.1 mmol × mm over a period of 0.2 s, i.e. two samples, occurred, and rejecting the block for that channel. Channels with data for less than 5 out of 12 blocks per condition were discarded. The minimum of 5 was chosen because it was found to coincide well with visual observation of individual data quality, thus providing an objective criterion. Note that the missing blocks rarely occurred subsequently. They tended to occur in random places across the testing session, thus allowing a reliable measure of learning across blocks. A baseline was established by using a linear fit over the 5 s time-window preceding the onset of the block and the 5 s window beginning 15 s after the end of the block. The 15 s resting period after stimulus offset was used to allow the hemodynamic response function (HRF) to return to baseline [10–46]. Analyses were conducted in MATLAB (version R2012b) with custom analysis scripts.

## Results

**Fig 3:**
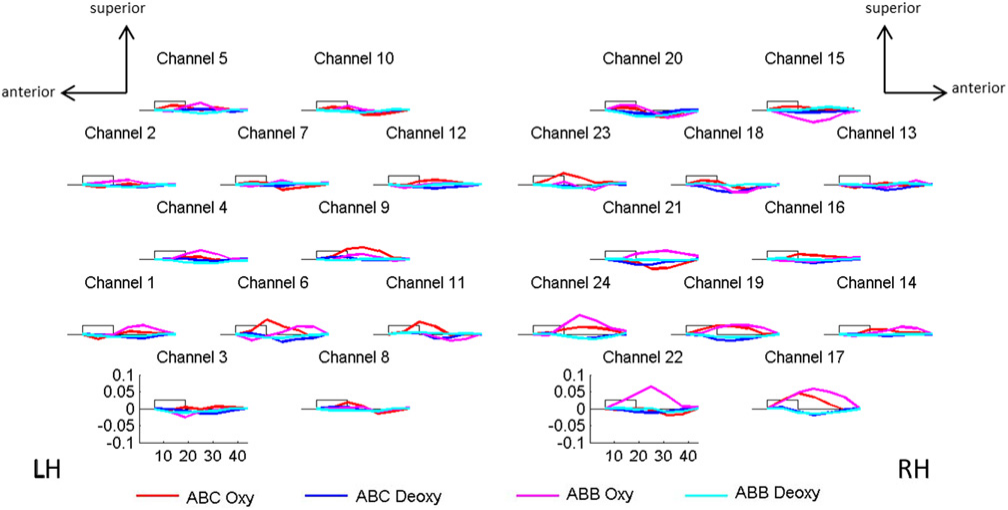
Grand average results. The concentration changes of oxy- and deoxyHb were averaged across the 12 blocks for each condition and for each channel. Numbers and location of channels correspond to the placement shown in Fig 2A. The x-axis represents time in seconds; the y-axis shows concentration in mmol x mm. The rectangle along the x-axis indicates time of stimulation. The continuous red and blue lines in the graphs represent oxyHb and deoxyHb concentrations, respectively, in response to the ABC grammar. The dashed magenta and cyan lines represent oxyHb and deoxyHb concentrations, respectively, in response to the ABB grammar. Note that the time line on the x-axis is of adequate duration given that it corresponds to the following sequence of events: 5s time-window preceding the onset of the block, block presentation (9.9s or 10.9s), and the following long inter-block silences (20 s or 25 s), thus a total duration ranging from 34.9s and 40.9s. This total period was divided in 7 time windows of equal length (~5.7s), and only the signal obtained between the time-windows 2 and 6 was used for statistical analyses, i.e. approximately between 5.7s and ~28.6s after block onset.

**Fig 4:**
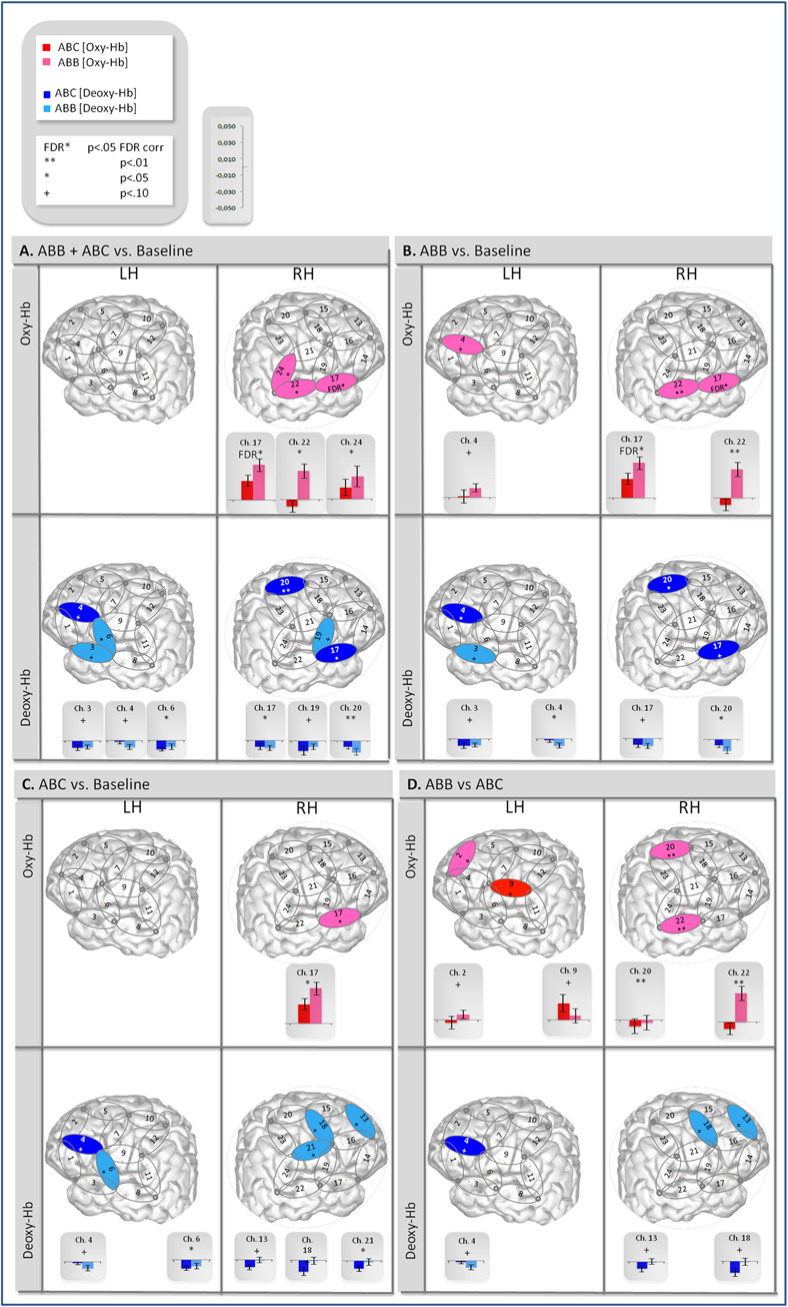
Schematic infant brain and bar graphs of oxyHb and deoxyHb concentrations (in mM.mm) for channels yielding significant t-test comparing responses to: ABB+ABC vs. baseline, (B) ABB vs. baseline, (C) ABC vs. baseline, (D) ABB vs. ABC. Significance levels for t-tests are indicated for each channel by *p-*values: ‘+’ marginally significant without correction for multiple comparisons;’**’ *p* < .01 significant without correction; ‘*’ *p* < .05 significant without correction; ‘FDR’ *p* < .05 significant after correction for multiple comparisons using the False Discovery Rate (FDR) [52], calculated using the FDR online calculator (http://sdmproject.com/utilities/?show=FDR). The FDR method is typically recommended to correct for multiple comparisons, such as multiple t-test analysis. Given a set of p-values, the FDR method returns p-values adjusted, controlling for the false discovery rate, i.e. the expected proportion of false discoveries amongst the rejected hypotheses.

The grand average results are presented in Fig 3. The figure shows the oxyHb and deoxyHb concentration changes averaged across all blocks of each condition and across all infants. The channel-by-channel *t-*test results for the mean oxyHb and deoxyHb changes are shown in Fig 4 for the following comparisons: the average of the two experimental conditions vs. baseline, ABB vs. baseline, ABC vs. baseline, and ABB vs. ABC.

As Condition Order did not have significant main effects or interactions in preliminary analyses, we collapsed over this factor in any further analyses. A first series of analysis of variance (ANOVA) with the within-subject factors of Grammar (ABB/ABC), Hemisphere (Left/Right) and Region of Interest (frontal/temporal) was run on average Hb concentration to evaluate whether the two grammars are processed differently overall, as in [10]. These ROIs were chosen given our probe configuration (Fig 2) in order to evaluate the responses in temporal areas (LH: channels 3, 6, 8, 11; RH: channels 17, 19, 22, 24) involved in auditory processing and in the frontal areas (LH: channels 1, 2, 4, 5; RH: channels 14, 13, 16, 15) involved in structural and more abstract processing in infants [35–53]. No structural brain scans were made of our participants so the relationship between probe locations, brain regions, and putative signal sources cannot be determined with certainty. Thus the assignment of probe channels to ROIs labeled as “Temporal” or “Frontal” is only approximate, especially for the developing brain. While our most anterior probe sites are clearly more frontal than our most posterior sites in each individual, there is considerable uncertainty about intermediate sites with respect to brain anatomy and ROIs. Thus the terms Frontal and Temporal ROIs should be interpreted in a relative sense. Furthermore, the presentation of our probe sites mapped onto a sample cortical surface (Figs 2A and 4) is for illustrative purposes and should not be interpreted as valid for any individual participant in the study.

Separate ANOVAs were conducted for oxyHb and deoxyHb. The ANOVA for oxyHb yielded a significant interaction between Hemisphere × ROI [*F*(1, 23) = 5.99, *p* = .02], as the activity in the RH was significantly greater in the temporal than in the frontal ROI, and temporal activity was significantly greater in the RH than in the LH (RH: frontal < temporal: *p* = .004; temporal ROIs: left < right: *p* = .01; these and all further post hoc *p*-values indicate results of Scheffe’s post hoc test for the relevant pairwise comparisons, unless otherwise specified). The interaction between Hemisphere × ROI × Condition was also significant [*F*(1, 23) = 4.71, *p* = .03] due to a stronger activation in the right temporal channels for the ABB condition (RH: ABB in temporal ROI > ABB in frontal ROI, *p* = .001; temporal ROIs: ABB right > ABB left, *p* = .003), and to several marginal tendencies (the ABB grammar yielded marginally greater activity than the ABC grammar in the left frontal ROIs, *p* = .08, and in the right temporal ROIs, *p* = .07; and the left frontal ROI was marginally more active than the right frontal ROI in response to ABB, *p* = .09). Moreover, the ANOVA for deoxyHb yielded a marginal Hemisphere × ROI interaction [*F*(1, 23) = 2.97, *p* = .09] due to significantly greater activation in the right temporal ROI over the left temporal ROI (p = .03). No other effects or interactions approached significance.

Together with the *t*-tests results (Fig 4), this first analysis reveals a somewhat different pattern than in [10]. Contrary to the generally stronger activation found for ABB over ABC establishing a clear general difference in the processing of the ABB and ABC grammars, we found no such effect. However, the greater response in the temporal as compared to the frontal channels was similar to the pattern observed in [10], suggesting that the stimuli were successfully processed by the auditory cortex in the current study, too. Additionally though, stronger responses were found in the right than in the left hemisphere, the opposite of what [10] found.

Importantly for the purposes of the current study looking at repetition suppression/enhancement effects, we ran a second ANOVA to examine the temporal dynamics of the responses to the ABB and ABC grammars during the course of the 12 consecutive blocks. This analysis also allowed us to test whether the apparent absence of repetition detection in the first overall analysis as well as the higher response observed in the right hemisphere could be understood in relation to potential differential increases/decreases in activation for the two conditions, occurring over time in response to the current material. In a similar analysis, [10] found that the perceptual detection of the ABB grammar was immediately observable in the first 4 blocks, and it further increased over the remaining blocks, whereas the response to the ABC grammar did not change throughout the experiment. Based on previous results regarding auditory perception in newborns [10–46], and the results of *t*-tests and the first analysis, a single ROI per hemisphere was used for this analysis: channels [1, 3, 8] corresponded to the left fronto-temporal area and channels [14, 17, 22] to the right fronto-temporal area (Fig 2A).

**Fig 5:**
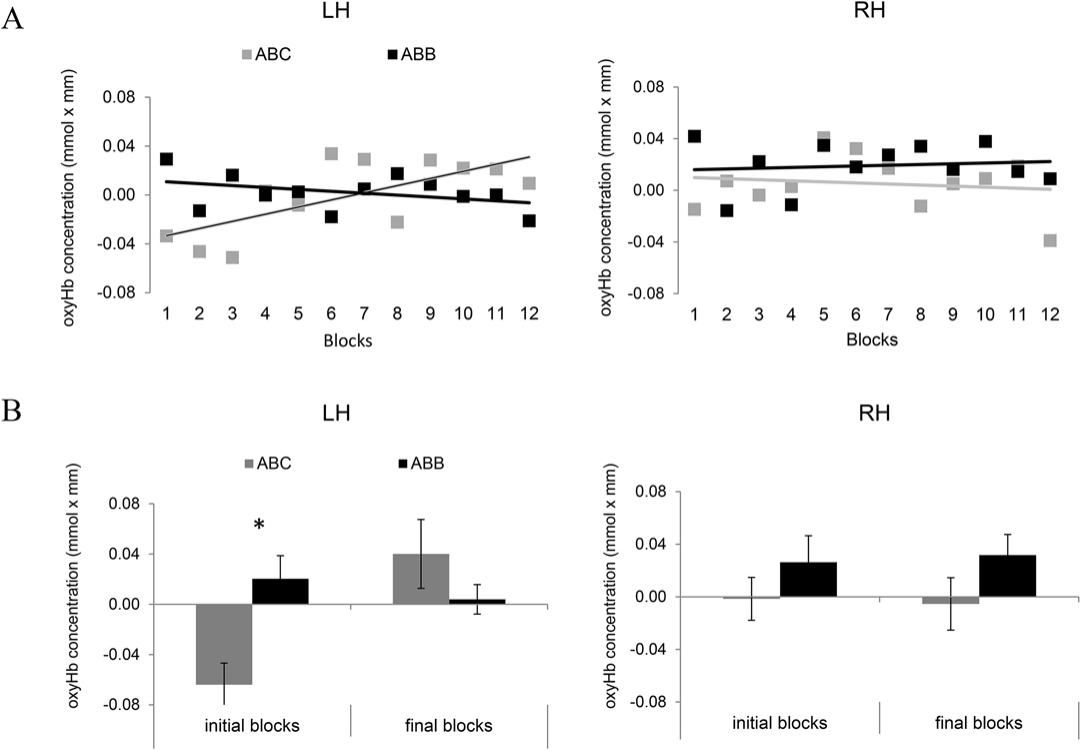
The time course of the responses for the two grammars in the left fronto-temporal ROI (channels 1, 3, 8) vs. the right fronto-temporal ROI (channels 14, 17, 22). The y-axis shows the average oxyHb concentration in mmol x mm. ABC is plotted in light-grey; ABB in black. (A) Linear regression lines of the oxyHb concentrations fitted on the data points provided by the 12 consecutive blocks for the two grammars, plotted on the x-axis. (B) The bars indicate the average oxyHb concentration for the first (“initial blocks”) and the last three blocks (“final blocks”).

We conducted ANOVAs on average Hb concentrations within the target ROI with the factors Grammar (ABB /ABC), Time (3 initial blocks/ 3 final blocks), and Hemisphere (LH/RH). For oxyHb, the ANOVA yielded a marginal main effect for Grammar [*F*(1, 23) = 3.80, *p* = .053], as ABB gave rise to a larger response than ABC. The Time × Grammar interaction was marginally significant [*F*(1, 23) = 3.63, *p* = .059], as initial blocks showed stronger activation for ABB than for ABC [*p* = .002], whereas activations to both grammars were equivalent in the final blocks [*p* = .2]. Importantly, the Time × Grammar × Hemisphere interaction was significant [*F*(1, 23) = 5.85, *p* = .017; Fig 5]. This was due to the fact that in the LH, the ABC grammar gave rise to an increasing response between initial and final blocks (*p* = .0005). Moreover, in the LH, there was a significantly stronger initial activation in response to the ABB over the ABC grammar (*p* = .002), but no such difference in the final blocks, where the activation for the ABC grammar had reached the same level as for ABB. In the RH, there was no difference between ABB and ABC in both the initial and the final blocks.

For deoxyHb, the same ANOVA also yielded a significant Time × Grammar × Hemisphere interaction [*F*(1, 23) = 6.51, *p* = .012]. This was due to ABB giving rise to a stronger initial deoxyHb response than ABC in the LH (*p* = .040), and ABC giving rise to a significant increase in the LH from initial to final blocks (*p* = .027). No other effects or interactions approached significance. In the RH, there was no difference between ABB and ABC in both the initial and the final blocks.

Overall, the oxy- and deoxyHb responses first show that, when averaged over all the blocks, the response to ABB is strongest in the right temporal ROI. Secondly, they show that in the left fronto-temporal area, the response to ABB is initially stronger than to ABC. Thirdly, again in the left fronto-temporal area, they establish that the response to ABC increases over time while a stable or slightly decreasing response to ABB is observed during the course of the experiment.

## Discussion

The goal of the present experiment was to assess the stimulus presentation conditions under which repetition enhancement [10] and repetition suppression effects might be observed in newborn hemodynamic activity in response to language stimuli. Specifically, we hypothesized that the repetition enhancement, rather than suppression, effect found in [10] was due to the high variability in the stimulus material used, requiring too long and effortful learning for neural habituation to occur during the time course of that study. Therefore, we tested whether the enhancement effect in [10] might be attenuated or reversed into a repetition suppression effect, as often found in the adult neuroimaging literature as a typical neural signature of learning. To this purpose, we used the same experimental paradigm as [10], but under conditions of relatively low stimulus variability, which might allow the newborn brain enough time and repetitions to show habituation. Accordingly, the current experiment tested the detection and learning of a repetition-based ABB grammar vs. an ABC random grammar, as in [10], but using a more redundant list of stimuli. Indeed, while [10] used 20 different syllables combined into 140 different sequences per condition, we used a list of 12 different syllables combined into 12 different sequences. Moreover, instead of interleaving the two conditions, they were presented consecutively. Lastly, note that although the total exposure to each grammar was reduced to 144 sequences in total per condition, as opposed to 280 in [10], each specific item is repeated more than in [10] (12 different times instead of only once), which should favor learning.

Our study has three major findings. First in the left fronto-temporal channels, we observed a stronger response to the ABB over the ABC grammars during the three initial blocks of the experiment. Previous findings have found a specific neural sensitivity to auditory and speech-processing and to adjacent repetitions at birth in this area. In line with previous NIRS [10–33] and behavioral [54] findings, this result may thus be interpreted as an initial left-lateralized advantage to the ABB grammar, and as an automatic detection mechanism specific to the structured ABB pattern over the random pattern in the newborn brain.

Secondly, we observed a strong hemispheric asymmetry, with the LH showing differential response dynamics, while activations in the RH, although relatively stronger overall and showing an advantage for ABB in the temporal area, remained constant in time for each condition. These lateralized responses could stem from the early left-lateralization of regularity extraction at birth, previously found in [46]. Alternatively, they might correspond to an advantage of the right hemisphere to process our stimuli in a constant and stable manner, with the left hemisphere requiring more neural effort to process the stimuli. This still suggests that changes in the response, which can be interpreted as learning, appear in the left hemisphere. Thus our findings contribute to the debate on the origins of the brain specialization and lateralization for learning regularities in speech.

Thirdly, and most importantly, we uncovered significant modulations in the time course of the responses to the two grammars, which, when compared with the results of [10], reveal the importance of variability/redundancy in the stimuli for inducing repetition effects. Here, after an initially small response to ABC, newborns’ left fronto-temporal cortex increased its activity over time to the ABC grammar, while the response to ABB, which was significant in the initial blocks, remained constant throughout the 12 blocks. A comparison with [10], where left fronto-temporal responses to ABB continued to increase during the course of the experiment while they remained constant for ABC, suggests that whether sequences were highly variable or very redundant had an impact on the direction of changes in neural response over time to the two grammars.

Our simpler and more redundant material resulted in two distinct learning curves for ABB vs. ABC, both of which were unexpected. First, in the ABB condition, contrary to our prediction, our redundant material did not reverse the direction of the repetition effect from enhancement to suppression. It only decreased the enhancement to a stable response. Further decrease in the complexity of the material might be necessary to fully reverse the effect. Indeed, the studies that observed repetition suppression in NIRS with young infants [8–9] all used classical habituation paradigms, where a single stimulus was repeated identically. Second, in the ABC condition, the left-lateralized increase does not confirm our initial hypotheses. At least two different interpretations are possible. One possibility, given that ABC sequences are composed of 3 instead of 2 different syllables, is that ABC sequences remained relatively less redundant in the material than ABB sequences, which may have yielded a time-increasing response to ABC. A second possibility is that item-based learning and memorization might have taken place in the present experiment, yielding an increased neural effort for the less structured ABC sequences.

More generally, whether gradual repetition effect may be observed varying from enhancement through stability to suppression as a function of gradually changing stimulus complexity, and specifically in response to the ABB grammar, is a question for further research. A related issue concerns the underlying processing mechanisms that stimuli of different complexity might trigger. Behavioral studies in artificial grammar learning indicate that variability in the input modulates the nature of the learning mechanisms elicited. When learners are confronted with linguistic material that contains constant as well as variable aspects, more variability typically leads to the extraction of the constant underlying regularity (in this case, the ABB rule) [30] while less variability favors memorization of specific items (i.e. individual sequences “mubaba”, “penana”) [31]. It may be the case that our simplified stimuli allowed automatic detection of the repetition within the ABB sequences, accounting for the initial advantage we observed for ABB over ABC, and converging with the original results of [10–33]. However, we might have decreased the variability in our stimuli to a point where abstract rule learning was not triggered. The extraction of the ABB rule from a smaller number of sequences than in [10] may have provided less opportunity for abstract learning processes to occur at the cognitive level, leaving the hemodynamic response stable over time.

It might be argued that other methodological differences could have contributed to the differences in results between the present study and [10], i.e. the blocked rather than interleaved presentation of the ABB and ABC blocks or reducing the duration of exposure to the ABC and ABB grammar from 280 to 144 sequences. Concerning the blocked vs. interleaved presentation of grammars, based on previous adult fMRI results where repetition suppression persisted even with intervening stimuli [54], it is unlikely that interleaved presentation alone could reverse the direction of the repetition effects. Moreover, the early automatic advantage for ABB sequences over ABC was replicated in the current experiment, suggesting that the basic detection of repetition did occur whether blocks were interleaved or not.

The reduced duration of exposure as compared to [10], is a consequence of our more redundant stimulus material. Given the low variability in our material, matching the length of exposure in our experiment to that of [10] would have induced the risk of a neural attenuation too strong to observe any statistically significant effects over the entire experiment. However, while overall exposure was indeed shorter, individual sequences were presented more frequently (12 times compared to once in [10]), giving more opportunity to learn at the item level. Moreover, the 144 sequences presented per condition were enough for the NIRS signal to increase over time in the ABC condition, suggesting that it would have been possible even under our stimulus presentation conditions to observe changes in neural response to the ABB grammar. Thus, the absence of a repetition effect in response to the ABB grammar cannot be entirely explained by the shorter learning period. Overall, we suggest that the differences between our findings and those of [10] stem mainly from our manipulation of stimulus variability.

The present results also contribute to a better understanding of how speech is processed and how regularities are learned at birth. First, the adjacent repetition pattern seems to be automatically detected, as was found consistently at birth using NIRS [10–33], and confirmed behaviorally [35]. Second, this advantage occurred in the initial blocks, suggesting that this detection mechanism is automatic, rapid, and perceptually based, allowing for the immediate discrimination of a local adjacent repetition carried by syllables. Moreover, differential response dynamics to the two grammars occurred only in the left hemisphere, especially in left infero-frontal and temporal channels, known to be responsible for speech and language processing, in line with a large body of evidence showing that dispositions for language learning are present in newborns (behaviorally: [55–60]); (NIRS: [8–46,61–63]) and even in fetuses (measuring heart rate and/or body movements: [64–65].

In conclusion, the present results provide new evidence of the modulation of repetition effects in speech processing in the newborn cortex. More specifically, they highlight the importance of an experimental variable, stimulus complexity, which impacts the amount of processing and learning required, and the presence or absence of the repetition enhancement effect in particular. Our findings further suggest that stimulus complexity may also be important for the direction of the repetition effect, although whether changes in stimuli variability might lead to a switch from enhancement to suppression remains to be established. Lastly, our results confirm that NIRS, a similar measure to fMRI, is not only more practical to use with young infants but can also be a relevant method for the investigation of the complex relationship between cortical activity and the functional level in these populations.
